# Clinical Characterization of a Table Mounted Range Shifter Board for Synchrotron-Based Intensity Modulated Proton Therapy for Pediatric Craniospinal Irradiation

**DOI:** 10.3390/cancers15112882

**Published:** 2023-05-23

**Authors:** William T. Hrinivich, Heng Li, Anh Tran, Sahaja Acharya, Matthew M. Ladra, Khadija Sheikh

**Affiliations:** 1 Department of Radiation Oncology and Molecular Radiation Sciences, Johns Hopkins University School of Medicine, Baltimore, MD 21287, USA; 2 The Johns Hopkins Proton Therapy Center, Johns Hopkins University School of Medicine, Washington, DC 20016, USA

**Keywords:** craniospinal irradiation, pediatric radiotherapy, proton therapy, pencil beam scanning, image-guided radiotherapy

## Abstract

**Simple Summary:**

Intensity modulated proton therapy provides unparalleled normal tissue sparing for the treatment of pediatric cancers such as medulloblastoma using craniospinal irradiation. However, proton delivery systems have a minimum deliverable energy and depth, requiring range shifter devices for the treatment of shallow targets and/or small anatomy. Standard range shifters are mounted to the treatment nozzle and introduce lateral scatter which, unfortunately, increases normal tissue dose. In this study we designed, manufactured, and characterized a range shifter board which rests directly under the patient during treatment to maintain a shallow treatment depth while minimizing lateral scatter, similar to previously proposed designs but optimized for our synchrotron-based delivery system. We present the dosimetric measurements we performed to characterize the device for treatment planning including severity of artifacts in on-board images. We compared the standard and range shifter board planning approaches in an anthropomorphic phantom and delivered the treatment plans to confirm the dosimetric differences. Finally, we compared treatment plans from two previously treated pediatric patients, demonstrating the ability to significantly reduce lung and kidney dose using this device compared to a standard range shifter. This device is now in routine use at our clinic.

**Abstract:**

*Purpose*: To report our design, manufacturing, commissioning and initial clinical experience with a table-mounted range shifter board (RSB) intended to replace the machine-mounted range shifter (MRS) in a synchrotron-based pencil beam scanning (PBS) system to reduce penumbra and normal tissue dose for image-guided pediatric craniospinal irradiation (CSI). *Methods*: A custom RSB was designed and manufactured from a 3.5 cm thick slab of polymethyl methacrylate (PMMA) to be placed directly under patients, on top of our existing couch top. The relative linear stopping power (RLSP) of the RSB was measured using a multi-layer ionization chamber, and output constancy was measured using an ion chamber. End-to-end tests were performed using the MRS and RSB approaches using an anthropomorphic phantom and radiochromic film measurements. Cone beam CT (CBCT) and 2D planar kV X-ray image quality were compared with and without the RSB present using image quality phantoms. CSI plans were produced using MRS and RSB approaches for two retrospective pediatric patients, and the resultant normal tissue doses were compared. *Results*: The RLSP of the RSB was found to be 1.163 and provided computed penumbra of 6.9 mm in the phantom compared to 11.8 mm using the MRS. Phantom measurements using the RSB demonstrated errors in output constancy, range, and penumbra of 0.3%, −0.8%, and 0.6 mm, respectively. The RSB reduced mean kidney and lung dose compared to the MRS by 57.7% and 46.3%, respectively. The RSB decreased mean CBCT image intensities by 86.8 HU but did not significantly impact CBCT or kV spatial resolution providing acceptable image quality for patient setup. *Conclusions*: A custom RSB for pediatric proton CSI was designed, manufactured, modeled in our TPS, and found to significantly reduce lateral proton beam penumbra compared to a standard MRS while maintaining CBCT and kV image-quality and is in routine use at our center.

## 1. Introduction

Craniospinal irradiation (CSI) is a common cancer treatment technique involving irradiation of the entire cerebrospinal fluid (CSF) in the brain and spine [1]. CSI is frequently employed to treat medulloblastoma and other cancers in pediatric patients, where smaller anatomy and increased potential for late treatment side effects create specific challenges compared to CSI in adults [2,3,4,5]. Intensity modulated proton therapy (IMPT) has been shown to provide unparalleled normal tissue sparing compared to photon-based CSI due to the finite proton range in tissue and ability to create modulated conformal treatment plans [5,6,7]. For modern pencil beam scanning (PBS) systems without additional collimation devices [8], lateral penumbra and the resultant conformity of the CSI plan are largely dependent upon the lateral pencil beam spot size [9,10,11]. The spot size in air tends to increase with decreasing energy (depth) until the minimum deliverable energy at the nozzle is reached, often approximately 70–100 MeV or 4–7 cm range in water [12,13]. For targets extending closer to the surface than this minimum depth, some form of compensator or range shifter is required at the nozzle to reduce the minimum proton energy and range. Machine-mounted range shifter (MRS) devices may be provided by the manufacturer to mount on the gantry nozzle or snout, which may or may not be adjustable to modify the air gap between the MRS and patient surface. In addition to proton range reduction, multiple-coulomb scattering (MCS) within the MRS introduces divergence in the pencil beam leading to a significant increase in spot size in the patient [14], which increases with air gap [11,12,13]. Range shifter solutions are frequently required for pediatric proton CSI to adequately cover shallow regions of the target, leading to increased lateral penumbra and dose to critical organs such as the lungs and kidneys.

Several groups have investigated methods of minimizing the air gap for this and other treatment sites to reduce penumbra. Both et al. generated universal bolus (UB) solutions to replace the MRS for a cyclotron-based system [15]. The UB consisted of a curved 5.5 cm water equivalent thickness (WET) surface and was mounted to the treatment couch in close proximity to the patient for cranial treatments. The UB significantly reduced spot size but did not rest directly on the patient surface and was found to introduce image artifacts due to the size and thickness. Lin et al. investigated various rangeshifter approaches for the same cyclotron system including a couch top bolus (CTB) consisting of a slab of fiberglass laminate placed directly under the patient with 7.35 cm WET, which could be employed for CSI treatments and demonstrated dosimetric benefits [13]. However, this system was also found to introduce significant CBCT artifacts and was not characterized by image-guided pediatric CSI. Michiels et al. demonstrated dosimetric benefits that could be obtained with custom patient-specific bolus (PSB) to eliminate the range shifter air gap for head and neck patients treated with proton therapy [16]. Kang et al. implemented a wax PSB approach to improve proton dosimetry for periorbital disease treatment; however, these PSB designs rested on the patient’s anterior surface and so are unsuitable for CSI without modification [17]. Wong et al. reported the use of a range shifter board (RSB) for pediatric whole lung irradiation (WLI) consisting of polymethyl methacrylate (PMMA) for use with a synchrotron-based PBS system, demonstrating significant reductions in heart and breast dose compared to photon irradiation [13]. Other commercial solutions for couch-top range shifter boards (RSBs) have recently become available but to our knowledge have not been characterized in-depth in the literature.

Furthermore, modern synchrotron-based IMPT delivery systems with dedicated nozzles now provide decreased spot sizes (sigma = 3–8 mm in air) and low minimum energies of approximately 70 MeV [18,19] which enables a reduction in RSB thickness compared to previously reported designs [13,15]. A thinner RSB may preserve on-board image quality while maintaining a sharp dosimetric penumbra, leading to overall improvements in treatment precision compared to current clinical standards. To this end, our clinic previously designed, manufactured, and commissioned an RSB for image-guided pediatric CSI using our synchrotron-based PBS system. In this report, we summarize this experience and provide comparisons with the manufacturer-provided MRS.

## 2. Materials and Methods

### 2.1. Proton Delivery System

The ProBeat compact-gantry system (Hitachi, Tokyo, Japan) at our institution is synchrotron-based, providing 98 discrete proton beam energies from 70.2 MeV to 228.7 MeV with in-air sigma at isocenter from 6.1 mm to 2.8 mm, respectively. We have three 360° gantries, which each have dedicated PBS nozzles with matched beams. The maximum field size is 30 × 40 cm^2^ at isocenter, and two nozzle-mounted X-ray panels enable orthogonal 2D and volumetric CBCT image-guidance. The depth of the distal 80% of the integral depth dose curve (R80) of the minimum energy is 3.9 cm in water, and the manufacturer provides a removable nozzle-mounted MRS manufactured from acrylonitrile butadiene styrene (ABS) resin with physical thickness of 3.9 cm and water equivalent thickness (WET) of 4.0 cm. Our nozzle is not moveable (i.e., no “snout”) with a constant gap between the MRS and isocenter plane of 39.1 cm. This system, including the MRS, was modeled in RayStation 10A SP1 (RaySearch, Stockholm, Sweden) treatment planning system (TPS), providing Monte Carlo dose calculation for treatment planning.

### 2.2. RSB Design

The RSB was designed as a rectangular slab of PMMA intended to be placed under the patient, on top of our ProPatient treatment couch (CIVCO, Coralville, IA, USA), similar to the PMMA RSB previously developed at St. Jude and employed in the study by Wong et al. for pediatric WLI [20]. The physical thickness of our RSB was designed to be 3.50 cm (WET of 4.07 cm). When placed on top of the treatment couch, which itself has a WET of 0.83 cm, the total WET of the couch + RSB is 4.90 cm, which exceeds the minimum required range reduction to treat the patient’s surface. This thickness was chosen to provide some design margin in case of density deviations in the PMMA and to ease manufacturing. The width of the board (left/right dimension) was chosen to be 46.99 cm (18.5″), which is slightly narrower than our treatment couch enabling visualization of the indexing locations and attachment of hand pegs. The total length of the board was 91.44 cm (36″), which is the maximum size of material that could fit on the vendor’s CNC router. With margins at each end of the RSB to accommodate the mask and indexing bar, this leads to a maximum useable treatment length of ~70 cm. Note that this RSB is too short for most adult CSI treatments, but the shorter length accommodates pediatric CSI treatments and limits the weight of the device to ease manipulation by staff, so it was deemed acceptable.

**Figure 1:**
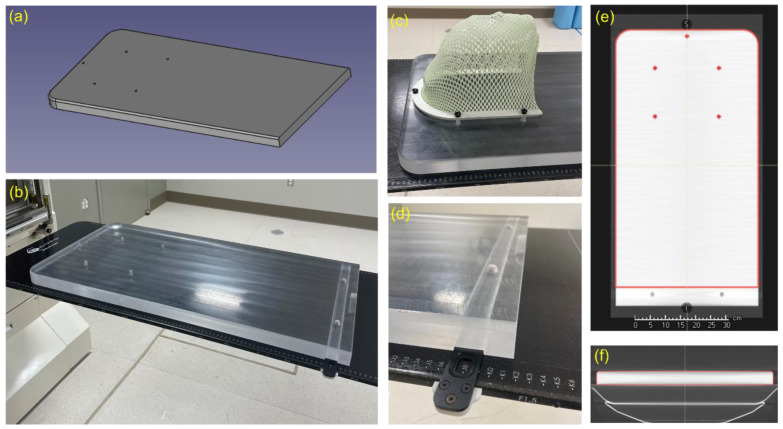
(**a**) CAD drawing of the RSB. (**b**) Photo of the RSB indexed to treatment couch. Note that the RSB has uniform thickness across all regions traversed by both cranial and spine fields. (**c**) Photo of mask attached to RSB mounting holes. (**d**) Photo of RSB indexing slot. This area cannot be used for treatment. (**e**,**f**) Screenshots of the RSB model aligned with CT scan of the RSB in the TPS. The model intentionally ends before the indexing bar location as a clear indicator of the usable RSB length within the TPS.

The RSB was designed to allow a moldable cushion and short mask (CIVCO, Coralville, IA, USA) to fasten directly to the board as required for CSI patient immobilization as shown in Figure 1c. This required the placement of five tapered holes for cushion and mask attachment similar to a commercial couch extension. A single slot was added near the inferior edge to enable indexing of the board with the couch as shown in Figure 1d. The top corners of the board were rounded to limit sharp corners near the treatment area. The RSB was modeled in FreeCAD V0.19 and manufactured off site by a vendor (.decimal Inc., Sanford, FL, USA).

### 2.3. Measurements

#### 2.3.1. Mechanical Verification

Following manufacture, the physical dimensions of the RSB were verified using calipers and a ruler. The RSB was placed on the treatment couch making use of the index bar and mask immobilization to test fit and attachment. The RSB was then scanned using a 64 slice Definition Edge Plus fan-beam CT scanner (Siemens Healthineers, Erlangen, Germany) with 120 kV tube potential and 2 mm slice thickness and imported in the TPS to verify dimensions and absence of internal defects.

#### 2.3.2. Relative Linear Stopping Power (RLSP)

**Figure 2:**
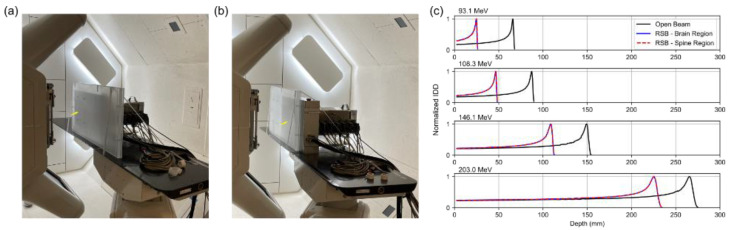
Experimental setup used to measure the RLSP of the RSB using the MLIC device. Two sets of measurements were performed in the head region (**a**) and spine region (**b**) indicated by the yellow arrows. (**c**) IDD plots of the open beam and beam after traversing the range shifter board for 4 beam energies.

A multilayer ion chamber (MLIC) (Hitachi, Tokyo, Japan) was used to determine the RLSP of the RSB by measuring the R80 for four pristine Bragg peaks (93.1 MeV, 106.3 MeV, 146.1 MeV, and 203.0 MeV), followed by the residual R80 after the same beams passed through the RSB. The “pull-back” was then computed as the difference between the two measurements. To ensure consistent density across the RSB, the pull-back was evaluated for two regions in the RSB approximately corresponding to the brain (Figure 2a) and spine (Figure 2b) treatment locations as shown in Figure 2. The average of the two measurements was used to compute the RLSP of the RSB (Figure 2c). The result of this measurement was then used to inform the design of a virtual model of the RSB including an accurate material override designed to match the physical dimensions and measured RLSP (Table 1).

#### 2.3.3. Output Constancy

As a test of the output constancy of the RSB setup compared to our standard calibration field, absolute dose measurements were acquired with a 30013 farmer chamber (PTW, Freiburg, Germany) for our standard reference spread-out Bragg peak (SOBP) at gantry angle of 0° and compared to the same field delivered at gantry angle of 180° through the ProPatient couch and RSB [21]. The reference SOBP field delivers a uniform physical dose of 2 Gy to a 10 × 10 × 10 cm^3^ cube with 20 cm range in water. A solid water phantom was used with the farmer chamber placed at isocenter such that the depth of the center of the chamber was at 15 cm WET for both the reference and test field.

#### 2.3.4. Image Quality

To evaluate image quality and artifacts introduced by the RSB, CBCT and kV images were acquired both with and without the RSB present. For CBCT, the CatPhan (The Phantom Laboratory, Greenwich, NY, USA) was set on top of the RSB enabling the evaluation of image quality. For the kV images, the Normi phantom (PTW, Frieburg, Germany) was used. The planar kV images were acquired with two tube potentials including 75 kV and 100 kV.

#### 2.3.5. Penumbra and Range in an Anthropomorphic Phantom

To quantify the difference in penumbra between the MRS and RSB approaches and validate the TPS model, two IMPT plans mimicking pediatric CSI spine fields were produced in the TPS delivered to the phantom containing radiochromic film (RCF) as follows. A CT scan of an anthropomorphic thorax SBRT phantom (CIRS, Norfolk, VA, USA) was acquired and imported in the TPS. Two copies of the scan were generated, where one was prepared using our standard couch model for use with the standard MRS, while the other was prepared using the RSB + couch model (no MRS). A 10 cm length segment of the spine structure in the phantom was contoured and used as the planning target volume. Both plans were optimized robustly with ±3 mm setup and ±3.5% and range uncertainties to deliver a uniform dose of 23.4 Gy[RBE] in 13 fractions to the target while minimizing the dose to the rest of the phantom.

The phantom was then set up at the proton gantry with a piece of RCF inserted in the center of the treatment field in the axial orientation and the bottom of the film abutting either the couch top for the MRS plan or RSB surface for the RSB plan. A single CBCT was acquired to align the phantom for each setup. Following each setup, a single fraction of the corresponding plan was delivered. Twenty-four hours later, the irradiated films were scanned using a 12000 XL flatbed scanner (Epson, Tokyo, Japan) in transmission mode at 75 dpi resolution with no color corrections [22]. The red channel of the image was converted into net optical density (OD), which was converted to a physical dose using a calibration curve derived from calibration films irradiated with protons in 50 cGy increments. Although RCF response has been shown to be strongly dependent on proton energy [23], no additional spectral or LET corrections were applied following dose conversion. Lateral profiles and depth dose profiles were extracted from the RCF and TPS centered on the target and used to compute lateral penumbra and range (R80).

### 2.4. Dose Evaluation in Pediatric CSI Patients

This retrospective study was approved by our institutional review board (IRB). In two previously treated pediatric CSI patients, the dosimetry was compared between the MRS and RSB approaches as follows. We generated two proton CSI plans for each patient using anonymized copies of the plan CT and contours, one with the MRS and one with the RSB, aiming to generate conformal treatment plans providing equivalent target coverage with prescription of 23.4 Gy[RBE] in 13 fractions. Our institution’s standard CSI beam arrangement was used for both plans, consisting of 2 posterior oblique beams (155° and 205°) to treat the brain and a posterior-to-anterior beam (180°) to treat the spine. A smooth PBS field junction technique was employed as described in previous studies, involving generation of a smooth dose gradient between cranial and spine fields in the region of overlap [9,10,24]. Note that although posterior oblique beams are used for the cranial fields, the RSB was designed with sufficient width ensuring that these beams also only traverse the regions of uniform RSB thickness including the field junctions to minimize any impacts on plan robustness. Plans were optimized robustly with ±3 mm of setup and ±3.5% range uncertainty. Following optimization, the treatment plans were normalized such that the coverage was the same (i.e., 95% of volume receiving prescription of 23.4 Gy[RBE]). The penumbra and mean dose (D_mean_) to the kidneys and lungs were computed and compared. Since the RSB has been commissioned and is in routine clinical use at our center, we also documented CBCT and kV image quality for a single pediatric CSI patient treated with the RSB present as a demonstration of clinical image quality.

## 3. Results

### 3.1. Mechanical Verification

All physical dimensions of the RSB matched the design as expected; the mask attachment and table indexing functioned correctly, and the CT scan also demonstrated correct dimensions and absence of any internal defects.

### 3.2. Relative Linear Stopping Power (RLSP)

The integrated depth dose (IDD) curves are shown in Figure 2 for four energies in the brain and spine regions. Measurement results demonstrated a mean pull-back of 40.72 mm. Comparing this to the physical RSB thickness of 35.00 mm, the RLSP was found to be 1.163 (Table 1). The RSB template was generated in the TPS by importing the CAD model using an in-house Python script. We tested overrides of the RSB using both water and PMMA as base materials with mass densities adjusted to 1.163 g/cc. In principle, setting the mass density equal to the RLSP is only applicable when using water as base material; however, in this case the PMMA base material provided an equivalent range. Since we expect proton scatter to be modeled more accurately when assigning PMMA as a base material, PMMA was selected for the final TPS model, although this difference has previously been shown to be small [25]. Finally, The RSB model was combined with the previously commissioned rectangular couch top model to ease import and alignment during planning and matching the intended clinical setup.

### 3.3. Output Constancy

The mean ± standard deviation of three charge measurements acquired using the reference field at gantry 0° and test field at gantry 180° passing through the RSB were 34.87 ± 0.03 nC and 34.80 ± 0.03 nC, respectively, corresponding to output constancy within 0.3%. Based on our monthly setup calibration factor and temperature and pressure corrections, these measurements corresponded to outputs of 0.998 cGy/MU and 0.995 cGy/MU, respectively.

### 3.4. Image Quality

**Figure 3:**
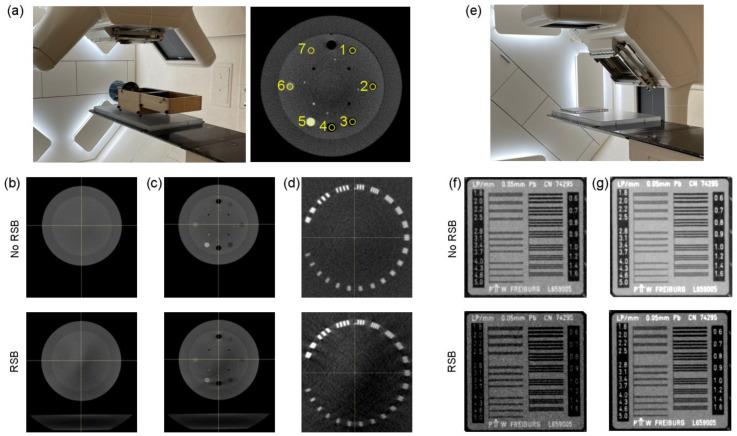
(**a**) Experimental setup of the CBCT evaluation (left) and indication of the 7 density plugs used for HU evaluation (right). (**b**–**d**) Phantom region used to evaluate HU heterogeneity (**b**), mean HU across inserts (**c**), and maximum visible line pairs (**d**). (**e**) Experimental setup of the kV planar imaging evaluation. (**f,g**) Line pair insert imaged using 75 kV (**f**) and 100 kV (**g**) tube potentials.

Figure 3 shows imaging results for both CBCT and kV images. All images had identical window and level between the “No RSB” and “RSB” conditions enabling visual comparison. The RSB introduced hardening artifacts in the CBCT images as shown in Figure 3b. However, spatial resolution is consistent between images acquired with and without the RSB. A similar effect is observed in the planar kV images in Figure 3f, where spatial resolution, as indicated by visible line pairs, was consistent. A comparison of metrics for the CBCT images is provided in Table 2. While there is an increase in HU heterogeneity and systematic decrease in HUs associated with the RSB, the overall image appearance and spatial resolution are maintained and so were deemed acceptable for patient setup.

### 3.5. Penumbra and Range Measurements in an Anthropomorphic Phantom

**Figure 4:**
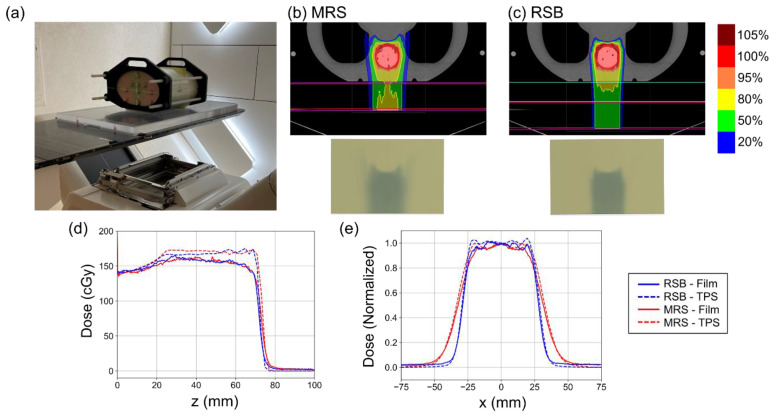
(**a**) Photo of the experimental setup used to verify the proton beam penumbra and range using the RSB. (**b**,**c**) provide isodose distributions on the top and images of the RCF following irradiation using the MRS and RSB approaches, respectively. (**d**) Plots of depth dose distributions as computed in the TPS and measured using film. Note the film under-response at the distal end of the proton beam where the energy spectrum is lower. (**e**) Plots of normalized lateral beam profiles computed in the TPS and measured using film.

The experimental setup, treatment plans, and results of RCF irradiation in the anthropomorphic phantom are shown in Figure 4. A reduction in penumbra associated with the RSB compared to the MRS is visibly apparent in the isodose distributions and irradiated films shown in Figure 4b,c. Figure 4d shows depth–dose profiles extracted from the dose distributions in the TPS and measured using RCF. RCF has been shown to under-respond to low energy protons such as those found near the Bragg peak [23], which we observed in the SOBP with greatest effect at the distal end of the beam profiles. Computed and measured ranges were found to be 71.9 mm and 71.1 mm for the MRS and 71.0 mm and 70.4 mm for the RSB indicating agreement between the TPS and measurement within the dose grid resolution (2 mm). Figure 4e shows lateral dose profiles extracted from the center of the target in the TPS and measured using RCF. Computed and measured penumbras were found to be 11.8 mm and 15.0 mm for the MRS and 6.9 mm and 7.5 mm for the RSB. Increased measured penumbra width was also associated with some under-estimation of dose at the beam edges using RCF leading to “rounded” profiles; however, overall beam profile and width agreed well with the TPS.

### 3.6. RSB in Pediatric CSI Patients

**Figure 5:**
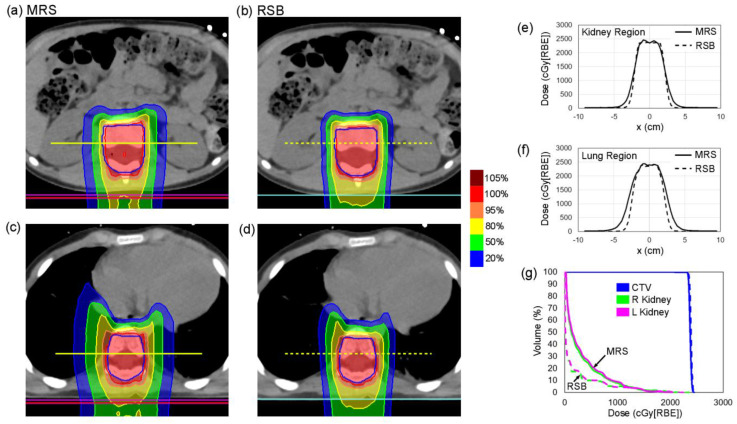
(**a**–**d**) CT and isodose distributions for CSI plans produced using the MRS and RSB approaches in a single patient at the level of the (**a**,**b**) kidneys and (**c**,**d**) lungs. (**e**,**f**) Lateral dose profiles extracted at locations indicated by yellow lines in (**a**–**d**) at the level of the (**e**) kidneys and (**f**) lungs. (**g**) Dose volume histogram (DVH) curves for the CTV and the left and right kidneys.

Figure 5 shows an example pediatric patient with an MRS based CSI plan (left: Figure 5a,c) and an RSB CSI plan (right: Figure 5b,d) demonstrating significant reduction in lateral penumbra provided by the RSB at the levels of the kidneys and lungs. Corresponding dose profiles across the center of the CTV at the level of the kidneys and lung are shown for the MRS (solid line) and RSB (dotted line) in Figure 5e,f. Dose volume histograms (DVH) for the example patient are provided in Figure 5g, demonstrating equivalent target coverage but significant reductions in kidney dose. Dose metrics associated with the two patients investigated are provided in Table 3, demonstrating reductions in mean kidney and lung dose of 57.7% and 46.3%, respectively.

**Figure 6:**
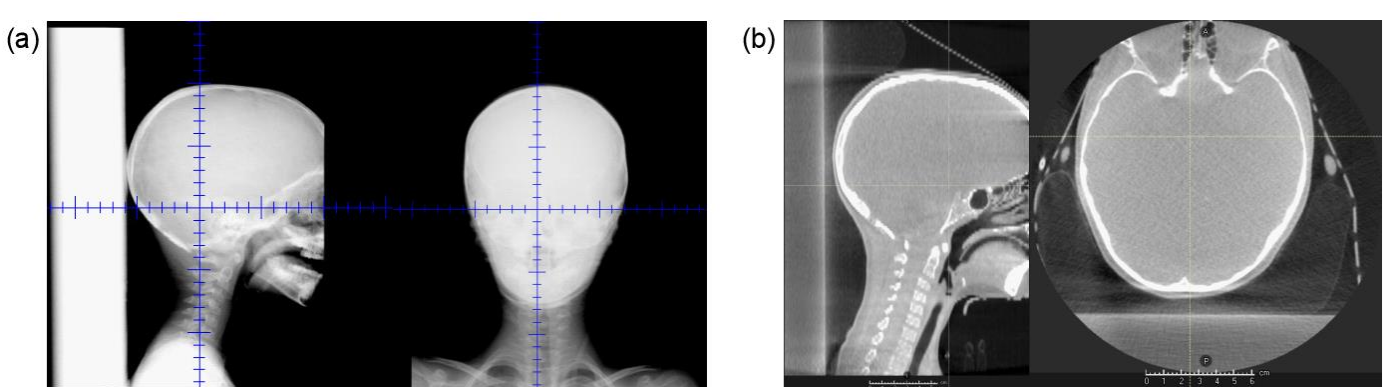
(**a**) Example (**a**) orthogonal kV images and (**b**) CBCT acquired using our on-board proton imaging system from a pediatric patient set up and treated with the RSB present.

Figure 6 shows (a) example planar kV images and (b) CBCT images acquired for a single pediatric CSI patient previously setup and treated with the RSB present, demonstrating preservation of image quality sufficient for patient visualization and setup. The RSB is visible in the sagittal images in both (a) and (b). In the axial CBCT image, it is apparent that there are visible beam hardening artifacts at the interface of the RSB and the posterior boney anatomy. However, these were not found to adversely impact assessment of patient setup.

## 4. Discussion

We have reported steps previously taken to develop, manufacture, and characterize a RSB for pediatric CSI using image-guided synchrotron-based PBS. We described design rationale, characterization of the dosimetric and imaging characteristics of the RSB to enable modeling in our TPS, results of validation measurements, and a preliminary demonstration of dosimetric improvements compared to the clinically standard MRS approach. By minimizing the air gap between the range shifter and patient surface, the lateral pencil beam spot size is reduced leading to significant improvements in lateral penumbra and ultimately the normal tissue dose. This principle has previously been demonstrated and implemented by several groups for various treatment sites [13,15,17]. In this report, we focused our design and characterization for pediatric CSI patients due to the smaller anatomy and increased potential for late effects due to low dose radiation; however, this type of device could be applicable to other treatment sites and patient populations making use of posterior PBS beams. The major limitation of the current device is the length, which limits the usable treatment length to ~70 cm. This length could be increased but would significantly increase the weight of the device making it more difficult to manipulate, which already represents a workflow challenge.

In addition to the technical considerations described in this report, our clinical commissioning process included many additional steps including developing and documenting simulation, treatment planning, and delivery workflows through a multi-disciplinary team including physicians, physicists, therapists, and dosimetrists. Briefly, our workflow involves simulating patients without the RSB present to eliminate any image artifacts that could impact our RLSP estimation based on CT. The patient headrest is positioned to avoid beams traversing the holes in the RSB, and patient height is evaluated to ensure the target will fit within the usable length of the board, avoiding the holes and indexing bar. Dosimetry and physics then insert the virtual model of the RSB at the time of treatment planning with the associated density overrides and remove the MRS from all treatment beams. This process is captured in our plan documentation and setup instructions at the machine, and a physicist is present on the first day of treatment to ensure the RSB is setup correctly. We also devised an approach where therapy will be prevented from loading the treatment fields if the RSB is omitted by differentiating the couch extensions used with the RSB or standard short mask couch setup in the oncology information system (OIS). Our proton gantries incorporate six degree-of-freedom robotic couches enabling rotational patient position corrections. To minimize range uncertainty due to beam obliquity through the RSB, we limit rotational corrections to ≤1° for patients treated using the RSB.

Soft tissue imaging is important in the setting of proton therapy as changes in the beam’s path can modify the proton dosimetry. As previously shown, different types of RS materials may have a different impact on the verification imaging quality [13] in particular on volumetric imaging such as CBCT. As the RSB is always present, its high density and thickness can lead to photon starvation which causes artifacts to degrade the image quality. We use daily CBCT and kV for all pediatric CSI patients, thus characterizing the imaging characteristics of our system in the presence of the RSB, specifically the spatial resolution and image heterogeneity. Using the Normi phantom and CatPhan, we determined that image quality preserved edges and is sufficient for patient set-up. Furthermore, we have not noticed any degradation of setup image quality in patients treated with the RSB since releasing it clinically.

The use of a similar 3.9 cm thick PMMA range shifter has previously been described for use with pediatric WLI [20]. We are utilizing the RSB clinically for all pediatric CSI patients and any pediatric patient requiring posterior beams where the use of the RSB may limit low dose to normal tissue. In pediatric patients where there is minimal lateral margin between the CSI spinal target and kidneys, this is especially important. Compared to previously published data, the penumbra achieved with the RSB is similar to what has been reported using passively scattered proton therapy (PSPT) with an aperture of 6–8 mm [24]. Similarly, the reported mean lung and kidney doses were similar to what has previously been reported using aperture-based PSPT [24]. Although the observed doses are not likely to lead to organ dysfunction, further normal tissue dose reductions are clinically desirable, especially in the pediatric setting [25,26,27]. Further improvements in lateral penumbra may be possible with additional collimation devices as explored by Winterhalter et al. [11] and may be the subject of future investigation.

Proton range shifting devices could change secondary dose contributions due to neutrons and other secondary particles. We did not report measurements of these dose components, which is a limitation of the current study. Kern et al. investigated the impact of range shifters and air gap on IMPT skin dose through measurements and Monte Carlo simulations and did find an increase in skin dose of up to 4% with the presence of range shifters with small air gaps compared to removal of range shifters and a larger air gap [28]. However, our clinical dose is computed using Monte Carlo [29], and, in the absence of the RSB, our supine CSI techniques involve irradiation through the existing couch top, itself acting as a thin range shifter. Given these considerations, we believe an increase in secondary dose is very small compared to the improvements in therapeutic dose conformity but could be the focus of future studies. Furthermore, previous investigators have demonstrated that PMMA exhibits fluorescence when irradiated by high-energy proton beams [30]. We have not investigated this phenomenon with our RSB devices, but it may be of interest for advanced beam monitoring or quality assurance applications in the future. Finally, in order to ensure clinical stability of the dosimetric RSB characteristics, we are continuing to investigate appropriate quality assurance approaches for this device including routine geometric and WET measurements.

## 5. Conclusions

We have summarized our clinical experience designing, manufacturing, and commissioning a custom RSB for image-guided proton CSI for pediatric cancer patients. The RSB was found to significantly reduce lateral proton beam penumbra compared to a standard MRS while maintaining CBCT and kV image-quality, thereby reducing normal tissue dose, and is now in routine use at our center.

## Figures and Tables

**Table 1:** MLIC measurement results.

Energy (MeV)	R80 (mm)			Pull-Back (mm)	
	Open Beam	RSB—Head	RSB—Spine	RSB—Head	RSB—Spine
93.1	66.50	25.10	25.36	41.40	41.14
108.3	88.08	47.06	47.33	41.03	40.75
146.1	150.76	110.11	110.27	40.66	40.49
203.0	267.97	227.71	227.92	40.26	40.05
Mean ± SD	-	-	-	40.84 ± 0.49	40.61 ± 0.46

**Table 2:** Results of CBCT imaging metrics without and with the RSB present.

	No RSB	RSB	Difference
Heterogeneity (HU_max_–HU_min_)	20.8	157.1	136.3
Mean HU Across Inserts	−50.5	−137.4	−86.8
Max. Visible Line Pairs (lp/cm)	7	7	0

**Table 3:** Dose metrics associated with the MRS and RSB CSI approaches in two patients.

	Patient 1		Patient 2		Mean	
	MRS	RSB	MRS	RSB	MRS	RSB
Kidney D_mean_ (Gy[RBE])	3.1	1.6	4.7	1.7	3.9	1.7
Lung D_mean_ (Gy[RBE])	4.3	2.1	3.9	2.3	4.1	2.2
Kidney Penumbra (mm)	13.0	7.0	15.0	7.0	14.0	7.0
Lung Penumbra (mm)	14.0	10.0	17.0	11.0	15.5	10.5

## Data Availability

The data are not publicly available due to privacy restrictions.
